# Rapid Non-Contact Detection of Chemical Warfare Agents by Laser Photoacoustic Spectroscopy

**DOI:** 10.3390/s24010201

**Published:** 2023-12-29

**Authors:** Luca Fiorani, Claudio Ciceroni, Isabella Giardina, Fabio Pollastrone

**Affiliations:** Diagnostics and Metrology Laboratory, Physical Technologies and Security Division, Nuclear Department, Italian National Agency for New Technologies, Energy and Sustainable Economic Development, Via Enrico Fermi 45, 00044 Frascati, Italy; claudio.ciceroni@enea.it (C.C.); isabella.giardina@enea.it (I.G.); fabio.pollastrone@enea.it (F.P.)

**Keywords:** chemical, biological, radiological, nuclear, and explosive (CBRNe) threats, chemical warfare agents, hazardous materials, laser photoacoustic spectroscopy, quantum cascade laser, multivariate calibration

## Abstract

Nerve agents have recently been used in battlefield operations, espionage wars, and terrorist attacks. These compounds, like some pesticides, cause organophosphate poisoning. The rapid, noncontact detection of a sarin simulant in the liquid phase has been demonstrated at the Diagnostics and Metrology Laboratory of the Italian National Agency for New Technologies, Energy and Sustainable Economic Development using laser photoacoustic spectroscopy, an infrared absorption technology. The first measurements, carried out with an experimental system based on a quantum cascade laser and developed for the assessment of food authenticity in the “fingerprint region”, show that a detection limit of one nanolitre is within the reach of the instrument when chemometric analysis is applied.

## 1. Introduction

According to the 2011 European Commission (EC) CBRN Glossary [1]: «CBRN is an acronym for chemical, biological, radiological, and nuclear issues that could harm the society through their accidental or deliberate release, dissemination, or impacts. The term CBRN is a replacement for the cold war term NBC (nuclear, biological, and chemical), which had replaced the previous term ABC (atomic, biological, and chemical) that was used in the fifties. “N” covers the impact of an explosion of nuclear bombs and the misuse of fissile material, “R” stands for dispersion of radioactive material e.g., by a dirty bomb», and CBRNe: «Is an acronym which includes beside CBRN explosive substances or events».

CBRNe materials can be weaponized (W-CBRNe) or nonweaponized (NW-CBRNe). W-CBRNe materials include weapons of mass destruction (WMD) and are intentionally used in criminal and terrorist activities. NW-CBRNe materials, also referred to as hazardous materials (HAZMAT), are linked to unintentional incidents or military operations as a secondary hazard. In both cases, serious consequences are expected for the affected population, such as intoxication, infection, irradiation, and, not least, the spread of panic.

Although the use of noxious gases has already been reported in historical times, the first large-scale use of chemical warfare agents (CWA) on the battlefield dates to World War I [2], infamously known as the Second Battle of Ypres (22 April 1915) in which the Germans used chlorine gas. Despite the Chemical Weapons Convention [3], their use has also been alleged in recent times. In addition, CWAs are now among the arrows in terrorists’ bows, as happened in the Tokyo subway [4]. Spy agencies are also suspected of employing them.

As mentioned, the occurrence of serious CBRNe accidents can also be unintentional, as the two following well-known chemical incidents demonstrate: (1) the Seveso accident, 1976 (no fatalities but hundreds of chloracne cases, decreased fertility, and increased risk of cancer and cardiometabolic outcomes) [5]; (2) the Bhopal Disaster, 1984 (about 4000 fatalities) [6].

Since 2017 [7], the Diagnostics and Metrology Laboratory (NUC-TECFIS-DIM) of the Italian National Agency for New Technologies, Energy, and Sustainable Economic Development (ENEA, Frascati, Italy) has been carrying out research in laser photoacoustic spectroscopy (LPAS) [8,9]. In an LPAS experiment, a laser beam is modulated at an audio frequency and directed into an acoustic cell, where it is absorbed by the sample under investigation. The heating and resulting increase in volume in the irradiated area causes a pressure wave that is detected by a microphone connected to a lock-in amplifier (LIA) synchronised with the modulator, which produces an output signal proportional to the absorption of the sample. Laser wavelength scanning is typically used to obtain the spectrum in the “fingerprint region”, a broad infrared (IR) band in which many organic compounds can be detected. The main advantage of LPAS over traditional lamp-based IR spectroscopy is the unparalleled power of the source, which offers rapidity, sensitivity, specificity, simplicity, repeatability, in situ measurement, uncomplicated sampling, ease of use, and cost effectiveness.

Although LPAS is most often applied to aeriform [10], recent research at NUC-TECFIS-DIM has focused on liquid and, more often, solid samples and a quantum cascade laser (QCL)-based LPAS system has been developed [11], upgraded [12], and patented [13]. The system has been applied to assess the safety and authenticity of fruit juices, oil, milk, pollen, rice (flour and cereals), seafood, and spices [14] In particular, it has been used to detect saffron fraud [15], to spot the production zones of various olive cultivars [16], and to identify oregano adulteration [17,18].

Chemometric techniques have largely proven to be a powerful tool to accurately extract valuable information from a set of seemingly indistinguishable spectra. Recent research at NUC-TECFIS-DIM has demonstrated that it is possible to obtain the multivariate calibration of the system to measure the amount of oregano in an unknown sample within minutes with a detection limit on the order of a few per cent [17].

The EC-funded MoSaiC project [19] is aimed at real-time monitoring of CBRNe events coupled with innovative sampling to improve (i) the dynamic mapping of threats and vulnerabilities, (ii) the response skills, including addressing CBRNe forensic priorities. The initiative adds abilities to existing detection, identification, and monitoring (DIM) platforms by working on a range of sensing capabilities and their integration. These capabilities include:

- Research into innovative and low-cost chemical and biological monitoring technologies, with a focus on the miniaturisation of existing technological solutions;
- Sampling technologies based on the concept of “smart swabs” to enable the rapid and nondestructive analysis of a sample for later laboratory analysis;
- Unmanned aerial vehicles (UAV) and unmanned ground vehicles (UGV) for surveillance and sampling;
- Real-time indoor and outdoor three-dimensional (3D) mapping and data processing of affected areas;
- Real-time four-dimensional (4D), i.e., 3D + time, visualisation for incident commanders, providing cost-effective monitoring capabilities, including data flow;
- Real-time communication between vehicles/sensors and command and control (C2) systems for decision support.

NUC-TECFIS-DIM contributes to the following three “key areas of innovation”:

1. Smart swab for chemical and biological sampling and monitoring. A sample is taken from a surface using a SERS-active swab to enable (i) near real-time nondestructive on-site detection and classification of a chemical or biological threat and, if positive, (ii) off-site analysis of the same sample in the laboratory using standard forensic methods. The swabbing, on-site analysis and storage of the sample can be performed by a small UGV equipped with a robotic arm, a Raman probe, and simple opto-electro-mechanical interfaces;
2. Laser-induced breakdown spectroscopy (LIBS) for stand-off monitoring of biological threats. A compact LIBS instrument for on-board UGV operation and stand-off detection of biological agents in aerosols and on surfaces has been developed with the ability to discriminate between interferants/substrates and with a processing algorithm to reduce false positives/negatives;
3. LPAS for chemical-threat monitoring. The instrument is based on a QCL, and the development of a LIA built on a field programmable gate array (FPGA). Chemometric techniques, principal component analysis (PCA), partial least square regression (PLS) or others, are used to analyse the acoustic signals generated by the thermal relaxation of the IR-laser absorption of the analytes. The sensor is compact and autonomous for operation on board robotic UGVs.

The results achieved to date in the latter “key area of innovation” i.e., a preliminary application to the detection of CWAs by LPAS combined with chemometrics [20] will be reported in this paper.

## 2. Materials and Methods

### 2.1. LPAS System

**Figure 1:**
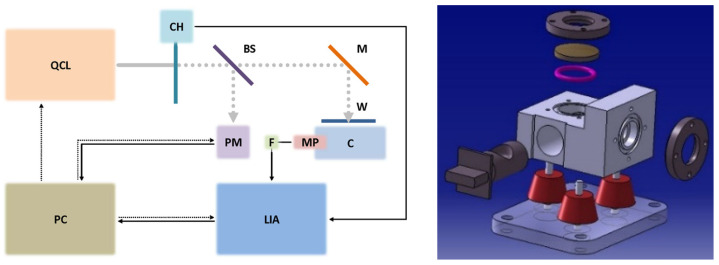
(**Left**): Block diagram of the LPAS system. BS: beam splitter, C: photoacoustic cell, CH: chopper, F: active low pass filter, M: mirror, MP: microphone, PC: personal computer, PM: power meter, W: window. Grey continuous line: continuous wave laser beam, grey dotted line: modulated laser beam, black continuous line: signal, black dotted line: control. (**Right**): exploded view of the photoacoustic cell: on the left, the drawer in which the sample holder is hollowed out (when inserted in the cell, the seal is ensured by a double o-ring): on the top, from top to bottom, window flange, window, and o-ring; on the right, microphone flange; on the bottom, three vibration dampening rubber feet.

The LPAS system has already been described in detail in this journal [17]. However, we recall here for the convenience of the reader:

- block diagram (Figure 1);
- main elements (Table 1);
- main specifications of the QCL, its core device (Table 2).

The diameter and height of the cell, made of black anodised aluminium, are 17.1 and 13.0 mm, respectively. The sensor developed at ENEA is unusual because it is designed for solid or liquid samples that are placed in a drawer that is inserted into the cell. The laser beam comes vertically from above (reflected by the mirror), passes through the window, and strikes the liquid or solid collected in the sample holder hollowed out in the drawer. For this reason, the laser beam does not exit the cell, and its power must be measured before entering, for example with a beam splitter and power meter as in this study. The numerical simulation, carried out with Ansys Sound 2023 [21] in collaboration with the University of Rome Tor Vergata [22], confirmed by the experimental measurement of the photoacoustic signal shows that the sensor sensitivity is maximum at 313 Hz (half width at half maximum about 600 Hz), far from the resonances; resonances occur at the expected frequencies, but the photoacoustic signal is higher at the low-frequency maximum.

The Knowles EK23024000 is an electret condenser microphone (output: analogue, directivity: omnidirectional). It costs a few tens of Euros and is small and light; it measures 5.56 mm × 3.98 mm × 2.21 mm and weighs 0.13 g. Despite this, it is extremely sensitive and low noise and has a robust construction to withstand harsh environmental conditions and high resistance to mechanical shock. Its sensitivity is better than −55 dB between 0.3 and 7 kHz (relative to 1.0 V/0.1 Pa), with a maximum of −48 dB at 4.5 kHz. The A-weighted noise (1kHz equivalent sound pressure level) is less than 26 dB.

**Figure 2:**
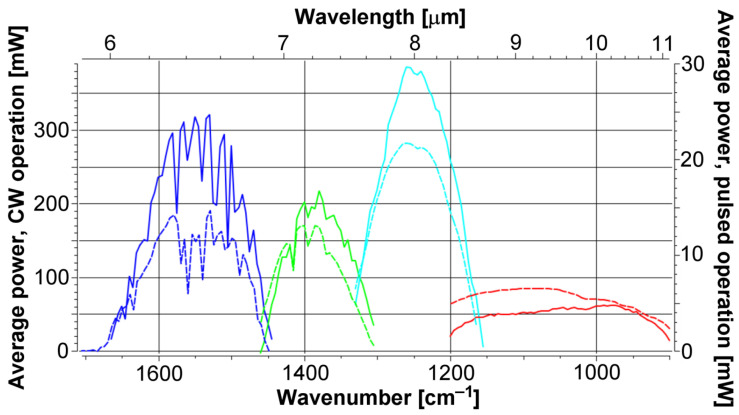
Spectral composition of the four QCL modules of which the DRS Daylight Solutions MIRcat-1200 is made. Continuous lines: continuous wave (CW) operation. Dashed lines: pulsed operation. Blue, green, cyan and red lines correspond to module 1, 2, 3 and 4, respectively.

The laser is modulated at 313 Hz with a mechanical chopper, as the DRS Daylight Solutions MIRcat-1200 cannot be electronically modulated at low frequencies. The beam splitter sends 18% of the laser beam to the power meter and 70% to the mirror (12% is absorbed by the beam splitter). Mirror reflectance and window transmittance exceed 97.5% and 92.5%, respectively. In this way, the average radiation power entering the detector is close to 20 mW. The radiation power by wavelength of the DRS Daylight Solutions MIRcat-1200 is shown in Figure 2.

The QCL wavelength is adjusted using an optical configuration patented by DRS Daylight Solutions. The gain chip is mounted between two collimating lenses. Light collimated to one side of the laser cavity is selectively reflected by a diffraction grating. As the grating is rotated, light of different wavelengths is fed back into the QCL, forcing it to emit a characteristic narrow linewidth.

### 2.2. Experiment Control

The experiment is controlled by a National Instruments (Austin, TX, USA) LabVIEW 2023 application developed at NUC-TECFIS-DIM. The LIA communicates through an Electronic Industries Alliance-recommended standard 232 (RS-232) [23] serial interface. The digital interface with the PC is accomplished via a universal serial bus (USB) virtual serial port converter. The data format complies with the Unicode transformation format, 8 bit (UTF-8) American standard code for information interchange (ASCII) [24]. The communication protocol is handled by a homemade LabVIEW driver. The QCL and power meter are connected to a USB hub which, in turn, is linked to the PC. The output file uses a plain ASCII format, which is easily readable and can be imported into MathWorks (Portola Valley, CA, USA) MATLAB 2023, Microsoft (Redmond, WA, USA) Excel 365, or other postprocessing applications. Data-file names are automatically generated to prevent accidental overwriting of previous measurements.

**Figure 3:**
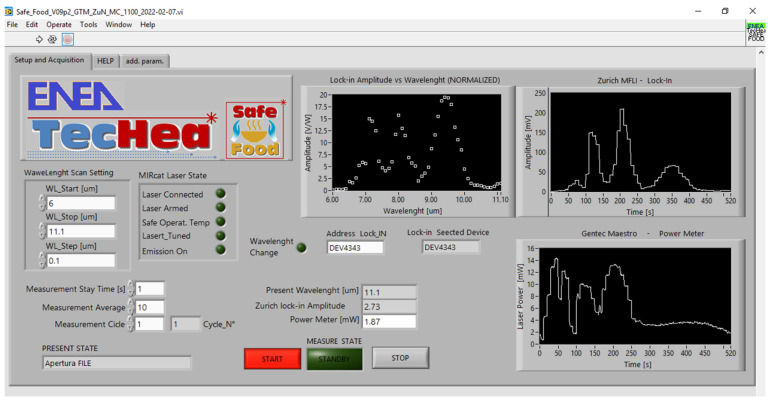
GUI of the photoacoustic laser system (developed in the ENEA-funded TecHea project [25]).

The graphic–user interface (GUI) (Figure 3) requires the three parameters of the spectrum (wavelength minimum, maximum, and step) and the number of measurements to be repeated at each wavelength (typical measurement duration: 1 s). Any point of the graphic of the photoacoustic signal vs. wavelength (LPAS spectrum) is calculated by dividing the average of the LIA voltages by the average of the laser powers. Each measurement cycle can be repeated automatically, and the cycle number appears on the screen.

### 2.3. Sample Preparation

As was mentioned in the introduction, in this study, we focused on CWAs that can be used on battlefields, in terrorist attacks, and in espionage wars. In many cases, a nerve agent was used. To avoid the risks associated with such compounds, simulants are usually used in the laboratory. A typical nerve-agent simulant is dimethyl methylphosphonate (DMMP) [26]. Since both compounds have a phosphorous–oxygen double bond, they have an absorption peak of about 7.9 µm, which makes them detectable with the photoacoustic laser system.

**Figure 4:**
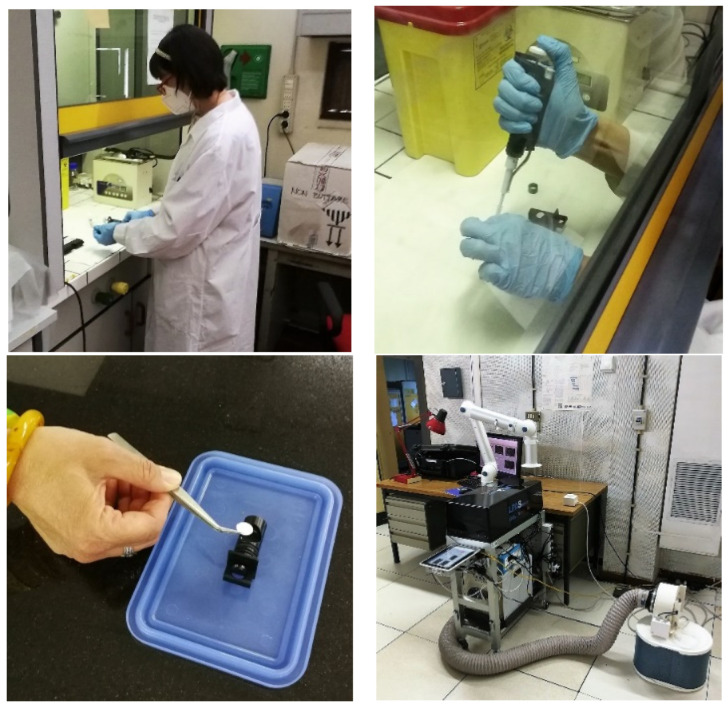
(**Top left**): handling of DMMP under a chemical hood. (**Top right**): preparation of a DMMP-soaked filter-paper disc; the LPAS system sample holder is visible on the table. (**Bottom left**): inserting the disc into the holder. (**Bottom right**): LPAS system with an aspiration system.

The scenario envisaged by the MoSaiC project involves a UGV travelling to the hot area and using a robotic arm to carry out a wipe test on a potentially contaminated surface by means of a paper smear, which is inserted into the photoacoustic cell via a motorised sample drawer. For this, samples were prepared by dropping 3 µl of DMMP onto 10 mm diameter filter-paper discs simulating paper smears (Figure 4) [27].

It is important to note that the MoSaiC scenario does not involve the LPAS of gases, but instead, the photoacoustic laser system is aimed at detecting a liquid nerve agent that has soaked a small disc of filter paper. Therefore, we are not concerned with the interference of atmospheric gases, nor can we translate the value of the detected volume of the liquid agent into its gas-phase concentration in ppm.

For comparison, samples were also prepared by dropping 3 µl of ethanol onto discs of the same type. Then, after thoroughly testing the photoacoustic laser system with a standard material (activated carbon), the LPAS spectra of DMMP, ethanol, and blank disc were measured.

### 2.4. Data Analysis

All spectra shown below were obtained as follows:

1. The QCL scanned the wavelengths from 7.00 to 10.00 μm with a step of 0.03 μm (101 wavelengths). Given that the typical measurement time is 1 s, 101 wavelengths are scanned in approximately 2 min;
2. The LIA and power meter measured the photoacoustic signal (V) and laser power (W), respectively. Each measurement took 1 s and was repeated 60 times (note that this corresponds to the acquisition of 60 raw spectra);
3. The 60 measurements of signal and power were averaged (note that this is equivalent to acquiring a single average spectrum);
4. In both cases (raw and averaged spectra), the LPAS signal (V/W) is given by the ratio of the signal and power measurements (thus normalising the photoacoustic signal to the laser power). In other words, the LPAS signal of the nth raw spectrum at a given wavelength is simply the ratio of the photoacoustic signal of the nth raw spectrum at that wavelength to the simultaneous laser-power measurement. Similarly, the LPAS signal of the averaged spectrum at a given wavelength is the ratio of the average of the 60 photoacoustic signals at that wavelength to the average of the 60 simultaneous laser-power measurements.

The acquisition of 60 spectra, which takes approximately two hours, serves to calibrate the instrument with a relatively large statistical sample and, thus, high accuracy. But once this calibration has been carried out, a DMMP concentration measurement is performed by acquiring a single spectrum, i.e., in approximately two minutes.

The averaged spectra were plotted and compared with standard data from the US National Institute of Standards and Technology (NIST) [28]. Finally, PCA and PLS [29] were applied to the experimental data using OriginLab (Northampton, MA, USA) OriginPro 2024 [30] and INRAE (Montpellier, France) ChemFlow 20.5 [31].

PCA is a statistical technique used to reduce the dimensionality of complex data sets while preserving important information. It identifies correlations in the data and transforms them into a new set of variables called principal components, which are linear combinations of the original variables. These components are ranked by the amount of variance they explain in the data, allowing the number of variables to be reduced while retaining as much of the original information as possible. In practice, our data set of 101 dimensions corresponding to the wavelengths is reduced to a data set of 3 principal components explaining almost all the variance thanks to routines available in OriginPro and ChemFlow. Naturally, the wavelengths around 7.9 µm are heavily weighted in the linear combination that produces the first principal component. PLS is a statistical method used to model relationships between sets of variables, which is not possible with PCA. It is often used in situations where there are several interrelated X variables (effects) and Y variables (responses). PLS attempts to find the linear combinations of the original variables (both effects and responses) by creating latent variables called X and Y components. These components are constructed to maximise the covariance between effects and responses. In general, PLS components generated from effects are similar but not identical to PCA components. Again, PLS is performed using routines available in OriginPro and ChemFlow.

## 3. Results and Discussion

**Figure 5:**
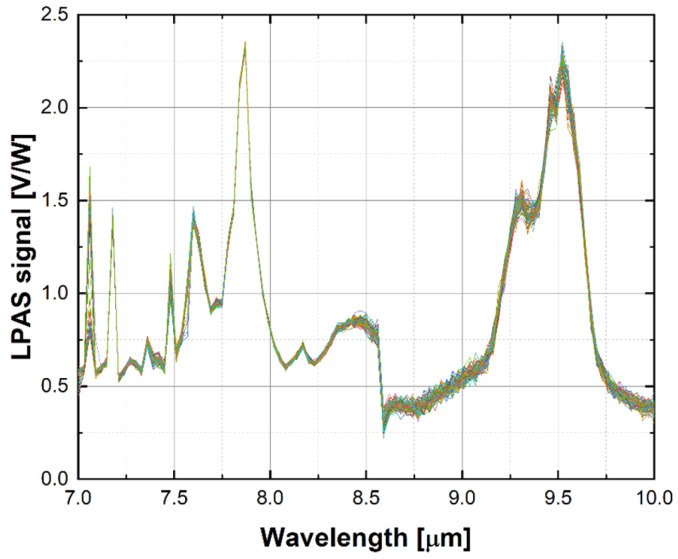
LPAS spectrum of DMMP (60 replicas).

**Figure 6:**
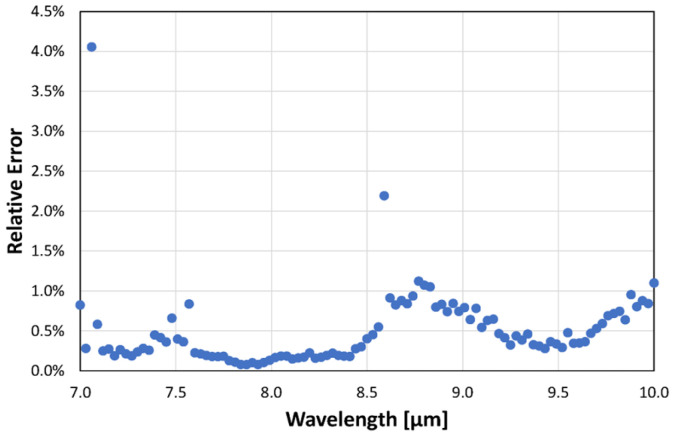
Relative error of the LPAS spectrum of DMMP (60 replicas).

The 60 raw spectra show good repeatability, as illustrated for example for DMMP in Figure 5. Looking closely at the graph, one can see that the LPAS signal is unstable at 7.1 µm, jumps at 8.6 µm, and is noisy between 8.6 and 10 µm. This behaviour is confirmed by the plot of the relative error of the LPAS signal (Figure 6), which is anomalously high at 7.1 and 8.6 µm and is relatively higher between 8.6 and 10 µm.

**Figure 7:**
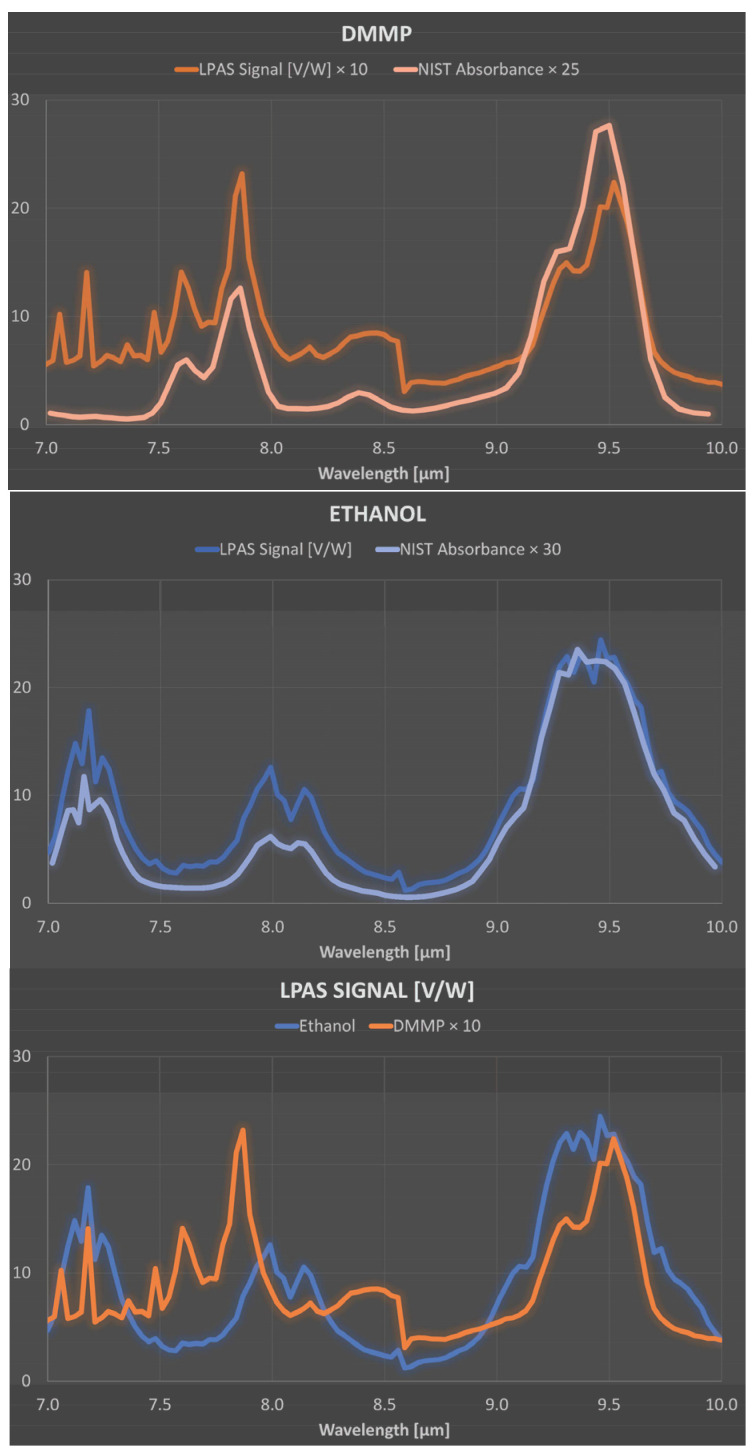
(**Top**): average spectrum of DMMP (LPAS signal and NIST absorbance). (**Middle**): average spectrum of ethanol (LPAS signal and NIST absorbance). (**Bottom**): average spectra of DMMP and ethanol. Note the difference in the signal scales.

**Figure 8:**
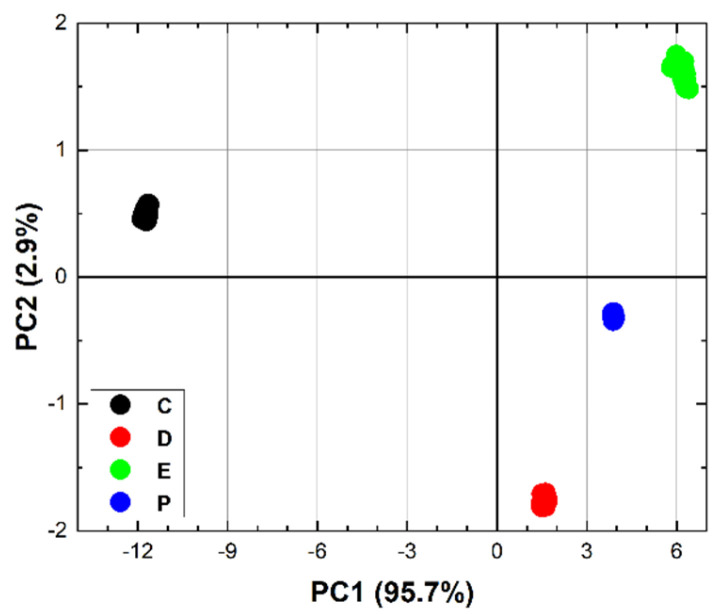
The 2D PCA of the 60 spectra of each sample C: activated carbon; D: DMMP; E: ethanol; P: blank disc).

Fortunately, the reason for the above behaviour at 7.1 µm, 8.6 µm, and between 8.6 and 10 µm is straightforward. Let us start with the higher relative error between 8.6 and 10 µm, which can be easily explained by the lower laser power in this spectral range. As for the instability at 7.1 µm, it is probably related to an observed temporal power fluctuation of the QCL, which has a noticeable drop in efficiency at this wavelength. The step at 8.6 µm is possibly related to a certain nonlinearity of the photoacoustic laser system. As we mentioned, the QCL consists of four modules, and, when switching from module 3 to module 4 at 8.6 µm, there is an evident decrease in laser power. But, the LPAS signal is reduced more than one would expect if it were proportional to the laser power. This behaviour can be related to the effectiveness of the source in heating the sample. It is reasonable to think that the material only heats up if the laser power exceeds a certain threshold and that, therefore, the effective power for heating between 8.6 and 10 µm is less than expected. In fact, as we shall see, this nonlinearity is barely perceptible for ethanol, which shows a much higher LPAS signal, linked precisely to a greater effectiveness of the source in heating the sample due, in this case, to the strong absorption of the latter compound. This is evident in Figure 7 (bottom) which shows the average spectra of DMMP and ethanol. The same figure also shows that the LPAS signal and the NIST absorbance are in good agreement for both DMMP (top) and ethanol (middle), and, very importantly for measurement purposes, these two compounds have very different spectra from 7.0 and 8.5 µm (bottom). It was, therefore, decided to apply chemometric analysis in this spectral range. The results (Figure 8) are very encouraging; PCA perfectly discriminates the four sample types already in the two-dimensional (2D) plots. When PCA is extended to 3D space, it explains 99.9% of the variance.

The next step is to apply PLS to calibrate the photoacoustic laser system. For this, samples at various DMMP concentrations are needed. Consequently, the following samples were prepared with the same procedure explained in the previous section:

- P: filter-paper disc;
- H: filter-paper disc soaked with 3 µl of water;
- 0: filter-paper disc soaked with 3 µl of DMMP (3000 nl of DMMP);
- 1: filter-paper disc soaked with 3 µl of DMMP diluted 10 times in water (300 nl of DMMP);
- 2: filter-paper disc soaked with 3 µl of DMMP diluted 100 times in water (30 nl of DMMP);
- 3: filter-paper disc soaked with 3 µl of DMMP diluted 1000 times in water (3 nl of DMMP).

And 10 spectra were measured for each sample from 7.00 to 8.50 μm with a step of 0.03 μm (51 wavelengths), corresponding to a total of approximately 10 min of operation.

**Figure 9:**
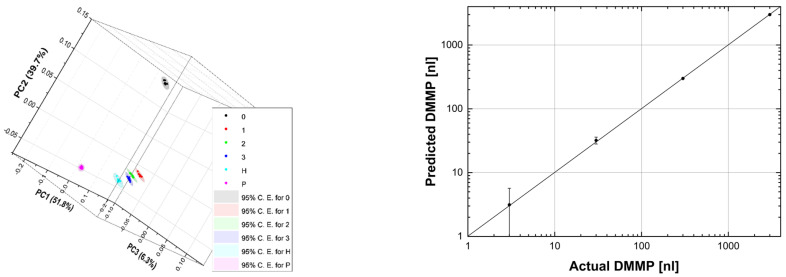
(**Left**): 3D PCA of the 10 spectra of each sample (C.E.: confidence ellipse). (**Right**): predicted DMMP vs. actual DMMP. The line is the identity function. In fact, the linear fit nearly coincides with it, having intercept: 0.08 ± 0.72; slope: 0.99987 ± 0.00054; R-squared: 1.0. The point corresponding to 0 actual DMMP cannot be plotted in the logarithmic graph but was included in all calculations.

Again, the PCA is comforting, as shown in Figure 9 (left): the point clouds, although not equidistant and along a straight line, are discriminated and arranged, as one would expect, from P to zero (going from right to left in the graph), passing, in order, through H, 3, 2, and 1. The explained variance is 97.8% with three principal components. These results were encouraging for the application of PLS, which indeed converged with six factors, thus explaining 99.0% of the variance for X (effects) variables and 99.9% of the variance for Y (responses) variables. Convergence was assessed by the root mean square of the predicted residual sum of squares (PRESS) [29]. Figure 9 (right) shows the difference between the predicted and actual DMMP, which is rather small. Note that a logarithmic scale has been used to display concentrations from 3 nl to 3000 nl, which makes the error bar of higher concentrations less noticeable. Table 3 summarises the PLS results. The maximum absolute difference, i.e., the absolute value of the difference between the observed value and the predicted value (residual), is 2.0 nl. The maximum statistical error is 4.0 nl. The absolute difference is less than the statistical error for all samples. The mean of absolute differences and statistical errors is 0.9 nl and 2.9 nl, respectively. With all that considered, a limit of detection (LOD) of 1 nl seems within the reach of the photoacoustic laser system, also bearing in mind that, as we saw in the first run of measurements, the number of spectra can easily be increased, thus reducing the statistical error. Of course, in preventing CBRNe events, the time factor is crucial, and the most convenient trade off might be to reduce the measurement time by accepting an increase in LOD. From this point of view, the photoacoustic laser system allows maximum flexibility, remembering that the measurement parameters can be chosen by the user.

## 4. Conclusions and Perspectives

### 4.1. Current Achievements

Having reached the end of this paper, it seems important to take stock of the situation. In the introduction, it was stated that the LPAS technique has the following advantages: rapidity, sensitivity, specificity, simplicity, repeatability, in situ measurement, uncomplicated sampling, ease of use, and cost effectiveness. It will then be useful to try to conclude where the system described here stands regarding these characteristics.

#### 4.1.1. Rapidity

As mentioned above, the measurement achieving LOD on the order of nl requires the acquisition of 10 spectra from 7.00 to 8.50 μm with a step of 0.03 μm (51 wavelengths). Remembering that the measurement at each wavelength takes 1 s, the whole operation lasts about 10 min. Compared to the usual chemical analyses performed in wet labs that can take hours if not days, the photoacoustic laser system can be described as rapid.

#### 4.1.2. Sensitivity

Frankly speaking, the fact of being able to detect a few nl of a nerve-gas simulant, moreover by sampling only 3 µl of liquid, with a relatively small filter paper disc, seems promising, bearing in mind that the system is in full development.

#### 4.1.3. Specificity

As candidly as we welcomed the sensitivity of the instrument, we must admit that there is still much to be done on this point, although the spectra of DMMP and substances not containing the phosphorous–oxygen double bond, such as ethanol, promise good specificity.

#### 4.1.4. Simplicity

The photoacoustic laser system is certainly complex from the point of view of mechanical, electronic, and optical realisation, adjustments, and chemometric analysis, but imagining that by automating the latter, the instrument, once fine-tuned, can be put into the hands of a not particularly well-trained person.

#### 4.1.5. Repeatability

This also needs further investigation. Nevertheless, not only are the replicas of the spectra performed subsequently clearly superimposable, but also the spectra acquired in the first (May 2023) and second (October 2023) run of measurements are perfectly consistent, which bodes well.

#### 4.1.6. In Situ Measurement

This is certainly a great advantage of the photoacoustic laser system. As we have mentioned, the project involves mounting the instrument in a UGV that travels to the hot zone to perform the CWAs presence measurements.

#### 4.1.7. Uncomplicated Sampling

Paradoxically, it was perhaps more complicated to prepare the samples in the laboratory and insert them into the instrument, adhering to a strict safety protocol, than to imagine its use in the field, which, as has been mentioned, will involve remotely piloted robotic arm insertion of the paper disc with which the wipe test can be performed.

#### 4.1.8. Ease of Use

This theme is related to the previous one. From the point of view of the operator driving the UGV and, with a few commands, ordering the execution of sampling and measurement, once well-engineered, the photoacoustic laser system could be within the capability of any user.

#### 4.1.9. Cost Effectiveness

Finally, it is necessary to reason about the cost effectiveness of the photoacoustic laser system. While a significant initial investment is needed to purchase the QCL (on the order of a few tens of thousands of Euros), the system is virtually capable of working for decades without consumables, spare parts, or maintenance work.

### 4.2. Future Perspectives

The most immediate future perspective that opens for the photoacoustic laser system is its deployment in terrorist-attack or battlefield scenarios, at least simulated ones, as envisaged by the project. For this, a compact version of the instrument of reduced size and weight compared to the cart-mounted instrument has already been planned. The reduction in size and weight will be possible, on the one hand, thanks to a new QCL already available at ENEA and, on the other hand, through the development in our laboratory of an FPGA-based LIA, which is much smaller than those available off the shelf. Of course, in the longer term, field deployment of a miniaturised and engineered photoacoustic laser system is desirable.

### 4.3. Open Issues

The interference of other substances in the scenario remains an important issue to be studied. In this preliminary research, we used DMMP and ethanol, two substances that have significant differences in their chemical bonding structures. But, what happens if organophosphate-based pesticides, compounds containing a phosphorous–oxygen double bond, are used at the terrorist-attack site or battlefield? Future lines of research will have to include studying the literature on common compounds found in real-world scenarios to see where there is overlap with nerve-agent absorption to find a region where IR spectra clearly discriminate their presence. Subsequently, it will be necessary to first perform laboratory experiments and then field tests of nerve-agent measurements in the presence of relevant interferents.

Another open question is the comparison between the LPAS system presented here and conventional gas-fingerprint spectroscopy of gases. At present, this is difficult because our work is aimed at the detection of liquid DMMP that has soaked a small disc of filter paper. Therefore, in the future, a new gas-spectroscopy cell should be implemented, perhaps with a device that heats the sample to assist in the vaporisation of the liquid chemical agent.

To make the photoacoustic laser system capable of real-time detection, chemometric analysis will have to be integrated into the software that controls the instrument and not as postprocessing of the spectra that are the actual output of the system.

One more instrumental improvement should be made in the direction of making the laser photoacoustic system more noncontact or even stand off, as is the case with LIBS and lidar instruments. Some attempts have been made [32], but the main difficulty is to abandon the cell.

It may also be useful to simultaneously measure the photoacoustic signal of both the unknown sample (paper disc soaked with an unknown chemical agent) and a blank sample (paper disc). In this way, normalisation of the former signal by the latter, rather than by the laser power, would take into account not only the laser fluctuation but also all those instrumental variables which are difficult to control, and which slightly alter the linear response of the system to laser excitation. As a result, the common mode noise should decrease. However, considering the increased complexity, size, and weight of the system on the one hand, and the fact that «linear multivariate regression may be able to correct the nonlinear deviations» [29], as confirmed by the results obtained here, this implementation does not seem to be a priority.

A further reduction in weight, size, and power consumption could be achieved by using miniaturised QCLs that emit a wavelength comb, i.e., a limited number of wavelengths rather than all the wavelengths in a specific band, as was the case with the QCL used in this study. With a judicious choice of wavelengths, it should be possible to intercept the key features of the nerve-agent absorption, even without the emission of a continuous spectrum, as is the case now. However, even when using the wavelength comb, the wavelength of the QCL radiation must be switched sequentially. With this significant improvement, it is reasonable to consider the possibility of fitting a photoacoustic laser system to a UAV in the future.

## Figures and Tables

**Table 1:** Main elements of the LPAS system.

Element	Manufacturer	Model
BS	Thorlabs (Newton, NJ, USA)	WG71050
C	ENEA ^1^	N.A.
CH	Thorlabs	MC2000B-EC
F	Hewlett-Packard (Palo Alto, CA, USA)	5489A
LIA	Zurich Instruments (Zürich, Switzerland)	MFLI
M	Thorlabs	PF10-03-M02
MP	Knowles (Itasca, IL, USA)	EK23024000
PC	AAEON (New Taipei City, Taiwan)	ACP-1106
PM	Gentec-EO (Quebec, QC, Canada)	UP12E-10S-H5-INT
QCL	DRS Daylight Solutions (San Diego, CA, USA)	MIRcat-1200
W	Thorlabs	WG71050-E4

^1^ The cell was designed at ENEA using a numerical simulation carried out with Ansys (Canonsburg, PA, USA) Sound 2023 [21] in collaboration with the University of Rome Tor Vergata [22].

**Table 2:** Main specifications of the QCL.

Wavelength range	6.0–11.1 µm
Linewidth	100 MHz
Wavelength accuracy	1 cm^−1^
Average power	60 mW
Power stability	3%
Spatial mode	TEM_00_
Beam divergence	4 mrad
Beam-pointing stability	2 mrad
Spot size	2.5 mm
Polarisation	Vertical 100:1

**Table 3:** PLS results.

Actual DMMP [nl]	Predicted DMMP [nl] (Average ± Statistical Error)	Absolute Difference [nl]
3000.0	2999.8 ± 3.0	0.2
300.0	299.1 ± 2.4	0.9
30.0	32.0 ± 4.0	2.0
3.0	3.1 ± 2.6	0.1
0.0	0.0 ± 2.5	0.0

## Data Availability

The data presented in this study are available on request from the corresponding author.
